# Taxonomic identification and lipid production of two Chilean *Chlorella*-like strains isolated from a marine and an estuarine coastal environment

**DOI:** 10.1093/aobpla/plt020

**Published:** 2013-03-20

**Authors:** Mariela A. González, Thomas Pröschold, Yussi Palacios, Paula Aguayo, Ingrid Inostroza, Patricia I. Gómez

**Affiliations:** 1 Departamento de Botánica, Facultad de Ciencias Naturales y Oceanográficas, Universidad de Concepción, Casilla 160-C, Concepción, Chile; 2 Department of Applied Ecology,University of Rostock, Albert-Einstein-Str. 3, D-18059 Rostock, Germany

**Keywords:** *Chlorella*-like strains, ITS secondary structure, light and transmission electron microscopy, lipid characterization, 18S rDNA sequences, taxonomic identification

## Abstract

The genus *Chlorella* was the first microalga to be massively cultured as food, feed and as a source of nutraceuticals. More recently, some species have been suggested as candidates for biodiesel production. One of the most difficult tasks in studying the systematics of green coccoids is the identification of species assigned to the genus *Chlorella*. In the context of several projects carried out by our research group we isolated two *Chlorella*-like strains from a marine and an estuarine coastal environment in Chile (Coliumo strain and Baker strain, respectively). The main objectives of this research were to identify these Chilean strains—at the species level—and determine and compare their lipid production when cultured under identical conditions. Cell size and shape, autospore number and sizes, and chloroplast and pyrenoid ultrastructure were considered as taxonomic descriptors, and 18S rDNA sequences and internal transcribed spacer ITS-1 + ITS-2 sequences and secondary structure were adopted as phylogenetic tools. The combined use of these morphological, ultrastructural and molecular attributes revealed that only the Baker strain belongs to the genus *Chlorella* (*C. vulgaris*), while the Coliumo strain corresponds to the recently amended genus *Chloroidium* (*C. saccharophilum*)*.* Lipid characterization of the biomass obtained from these strains showed that *Chlorella vulgaris* (Baker strain) appears to be suitable as a raw material for biodiesel production, while *Chloroidium saccharophilum* (Coliumo strain) would be more appropriate for animal nutrition.

## Abstract (precis)

This paper emphasizes the value of knowing the correct identity of microalgal strains that may have biotechnological potential. Here, two Chilean small green coccoid referred as *Chlorella*-like strains were identified using a polyphasic approach. Only one of them corresponded to the genus *Chlorella (C. vulgaris)*; the other belonged to the recently amended genus *Chloroidium (C. saccharophilum)*. Lipids characterization of the biomass obtained from these strains showed that *Chlorella vulgaris* (Baker strain) appeared to be suitable as raw material for biodiesel production, while *Chloroidium saccharophilum* (Coliumo strain) would be more appropriate for animal nutrition.

## Introduction

Green algae represent one of the main taxonomic groups from which oleaginous strains have been identified. Two main applications of lipid-rich strains have been proposed: as raw material for biodiesel production (Hu *et al*. 2008; Converti *et al*. 2009; Rodolfi *et al*. 2009; Lu *et al*. 2010) and as essential fatty acid sources for animal and human nutrition (Spolaore *et al*. 2006).

The genus *Chlorella* encompasses spherical or ellipsoidal non-motile green cells that produce autospores, and inhabit freshwater, soil and marine habitats (Kómarek and Fott 1983). Its commercial potential has been considered since 1960, being the first microalga to be mass cultured for food, feed and as a source of nutraceuticals (i.e. vitamins and antibiotic substances) (Becker 1986; Borowitzka 1988, 1999; De Pauw and Persoone 1988). More recently, it has also been suggested that they are good candidates for fuel production (Li *et al*. 2007; Converti *et al*. 2009; Mata *et al*. 2010).

The insufficiently distinctive characters visible under the light microscope have forced phycologists to use additional criteria to decide whether any spherical or ellipsoidal green cells that produce autospores do or do not belong to the genus *Chlorella*. Thus, attributes such as cell wall ultrastructure (Yamada and Sakaguchi 1982) and chemical composition (Takeda 1991), pyrenoid ultrastructure (Ikeda and Takeda 1995), comparative biochemistry and physiology of taxa (Kessler and Huss 1992) and, more recently, DNA sequences (Huss and Sogin 1990; Aráoz *et al*. 1998; Cozzolino *et al*. 1999; Huss *et al*. 1999; Darienko *et al*. 2010; Bock *et al.* 2011; Pröschold *et al*. 2011) can be extremely useful for correct classification.

Based mainly on biochemical and molecular data, Huss *et al.* (1999) updated the genus, confirming its already suspected polyphyly (Huss and Sogin 1990). Of the 19 taxa studied and assigned to *Chlorella* (based on Kessler and Soeder 1962), they recognized only four (*C. vulgaris* Beijerink-type species, *C. lobophora* Andreyeva, *C. sorokiniana* Shihira & Krauss and *C. kessleri* Fott & Novakova) as ‘true *Chlorella*’. Later on, Krienitz *et al.* (2004), utilizing the 18S rDNA and internal transcribed spacer ITS-2 sequences, transferred *C. kessleri* to a newly described genus, *Parachlorella*, leaving the true *Chlorella* species reduced to the three remaining taxa cited above. Other common attributes shared by these taxa are glucosamine as the predominant compound of the cell wall, a double thylakoid membrane traversing the pyrenoid matrix and an absence of secondary carotenoids (Huss *et al.* 1999). All the other *Chlorella*-like taxa studied by the above authors, including the ellipsoidal ones (Darienko *et al*. 2010), have been transferred to other genera [*Chloroidium* (Darienko *et al*. 2010), *Watanabea* (Hanagata *et al*. 1998), *Mychonastes* (Kalina and Punčochářová 1987)] or to new created genera [*Auxenochlorella* (Kalina and Punčochářová 1987) and *Parachlorella* (Krienitz *et al*. 2004)]. More recently, and based mostly on molecular data, Pröschold *et al*. (2011) and Bock *et al*. (2011) increased the species number of the ‘true *Chlorella*’ to 14.

In the context of several projects carried out by our research group (FICOLAB, www.ficolab.cl), we isolated two *Chlorella*-like strains, which are currently maintained and incorporated into the Culture Collection of Microalgae of the Universidad de Concepción, Chile (CCM-UDEC). In 2005, we isolated a green coccoid strain from a phytoplankton sample originating from Chilean coastal water, close to a natural population of the clam digger, *Ensis macha* (Coliumo Bay, Bío-Bío Region). More recently, in 2008, we isolated another strain, with similar morphological attributes under the light microscope, from the Baker River estuary, Aysén Region, a characteristic turquoise-blue coloured river of Chilean Patagonia.

The aims of this research were (i) to identify taxonomically, at the species level, the Coliumo and Baker strains by means of ultrastructure and molecular attributes and (ii) to determine and compare the growth parameters and lipid production of both strains, grown under identical culture conditions.

## Methods

### Strain identification

#### Sampling and isolation of strains

The strains were collected from either coastal marine or estuarine water using 33-µm-diameter nylon mesh. The marine strain (here, the Coliumo strain) was collected at a depth of ca. 10 m in April 2005, close to a natural population of the clam digger *E. macha*, located in Coliumo Bay (36° 32′S; 72°57′W). The estuarine strain (here, the Baker strain) was collected in September 2008, from the estuary of the Baker River (47°47′43″S; 73°34′57″W) in the Aysén Region, Southern Chile.

Unialgal cultures of the strains under study were obtained by micropipetting and/or streaking across agar plates (Hoshaw and Rosowski 1973), inoculated in tubes with f/2 medium and successively transferred to and maintained in Walne medium (Andersen 2005). Under the latter medium, the strains have been maintained at a photon flux density (PFD) of 15–20 µmol m^−2^ s^−1^, a temperature of 15 ± 2 °C and a photoperiod of 16 : 8 h (light : dark). Both strains were incorporated into the Culture Collection of Microalgae of the Universidad de Concepción, Concepción, Chile (CCM-UDEC): the Coliumo strain as CCM-UDEC 143 and the Baker strain as CCM-UDEC 221.

#### Observation of strains under light and transmission electron microscopy

Live cells were examined under an Olympus CX31 microscope fitted with bright-field and phase-contrast optics. The photomicrographs were taken with an Olympus C3040 camera attached to the microscope.

For transmission electron microscopy (TEM), culture material of the Coliumo strain was prepared following the protocol of Moestrup and Throndsen (1988). Basically, the cells were harvested with gentle centrifugation and fixed for 1 h in an equal volume of 2 % glutaraldehyde and 0.1 M buffer cacodylate at neutral pH. In order to reduce the osmotic changes, 0.2 M sucrose was added. After washing the samples three times with decreasing sucrose concentrations (0.2, 0.1 and 0.05 M) for 2 h, the cells were treated for 3 h in a mix of 2 % osmium acid and 0.1 M buffer cacodylate, followed by a wash in sacarose buffer and dehydrated in a series of ethanol concentrations (30, 50, 70 %, and twice in absolute ethanol) for 15 min each, then transferred to propylene oxide for 5 min and kept overnight in a mix of 1 : 1 propylene oxide and Spurr. Finally, the samples were polymerized in Spurr at 60 °C. For the Baker strain, the protocol described by Rascio *et al*. (1980) was used, since no good results were obtained with the above-described methodology. Ultrathin sections were obtained on a Sorvall MT 5000 (Du Pont) ultramicrotome, collected on slot grids and double-stained with uranyl acetate and lead citrate (Reynolds 1963). Observations were carried out with a JEOL JEM 1200EX-II transmission electron microscope. The photomicrographs were taken with a camera attached to the microscope.

### DNA extraction, amplification and sequencing

#### For the Baker strain

Total DNA was extracted according to the methodology described by Gómez and González (2001). The ITS region was amplified with the primers AB28 (5′-ATATGCTTAAGTTCAGCGGGT-3′) and TW81 (5′-GTTTCCGTAGGTGAACCTGC-3′), as in Goff *et al*. (1994), and the ARNr 18S gene with the primers P2 (5′-CTGGTTGATTCTGCCAGT-3′) and P4 (5′-TGATCCTTCYGCAGGTTCAC-3′), as in De Wever *et al*. (2009). The amplified fragments were purified using the ‘Wizard SV and PCR Clean-Up System’ kit (Promega). The purified fragments were cloned utilizing the PGEM-T Easy Vector kit (Promega). Plasmids with the cloned sequences were sent to Macrogen (Korea) for automated sequencing.

#### For the Coliumo strain

Genomic DNA was extracted using the Dneasy Plant Mini Kit (Qiagen GmbH, Hilden, Germany) following the instructions provided by the manufacturers. The SSU and ITS rDNA were amplified according to Luo *et al*. (2006) using the Taq PCR Mastermix Kit (Qiagen GmbH, Hilden, Germany) with the primers (EAF3: 5′-TCGACAATCTGGTTGATCCTGCCAG-3′ and ITS055R: 5′-CTCCTTGGTCCGTGTTTCAAGACGGG-3) published by Pröschold *et al.* (2001) using the following PCR cycle programme: 96 °C for 5 min, 30 cycles (96 °C for 1 min, 55 °C for 2 min and 68 °C for 3 min) and 68 °C for 10 min.

### Alignment and phylogenetic analyses

The SSU rDNA sequences of both strains were incorporated into an alignment of all representatives of the Trebouxiophyceae and aligned according to their secondary structures. As a template, the secondary structure of CCAP 248/5 *Micractinium pusillum* was used (see fig. S1 in Luo *et al*. 2006). This data set (36 taxa, 1747 bp) was analysed by PAUP v 4.0b10 (Swofford 2002). To test which evolutionary model fitted best for the phylogenetic analyses, the log-likelihood values of 56 models were estimated by PAUP, and the best model was calculated by Modeltest 3.7 (Posada and Crandall 1998; Posada and Buckley 2004) using the Akaike information criterion. The phylogenetic tree was inferred by maximum likelihood (ML) using PAUP and TIM+I+G model, which was estimated by Modeltest as the best model. To test the robustness of the phylogenetic tree, bootstrap analyses [neighbour-joining using the TIM+I+G model (1000 replicates), maximum parsimony (1000 replicates) and ML using the TIM+I+G model (100 replicates)] were conducted. The secondary structures of both ITS-1 and ITS-2 strains were performed using mfold (Zuker 2003) and illustrated by the program LoopDloop (Gilbert 1992).

### Culture conditions and analytical methods for lipid quantification

#### Growth conditions and biomass production

The strains were grown in 1 L of Walne medium at 20 ± 2 °C, salinity of 35 psu, 200 µmol m^−2^ s^−1^ PFD and 16 : 8 h (L : D) photoperiod. The cultures were continuously aerated with 0.22 µm pore-filtered air. All cultures were initiated by the inoculation of each flask with exponentially growing cells (previously acclimated to the experimental conditions for 15 days) at a density of 10 000 cells mL^−1^. Four replicates of each strain were established.

Growth was monitored over 17 days by cell counting in a Neubaüer chamber every other day. The maximum growth rate, *k*_max_ (divisions day^−1^), was determined during the logarithmic growth phase as in Guillard (1973). At Day 17, the cell density was also assessed to estimate the total lipids per cell.

The algal dry weight was determined after 17 days of cultivation by filtering 10-mL aliquots through pre-dried and weighed Millipore filters of 0.45 µm pore size, washing the filtered aliquots with 0.5 M ammonium formiate and drying them at 80 °C until they reached a constant weight.

### Lipid analyses: extraction and quantification of total lipids

Biomass from each replicate and strain was harvested by centrifugation at 3500 rpm for 5 min at 4 °C and dried at 37 °C for 2 weeks. Thirty milligrams of dried biomass were used to determine the total lipid content according to Bligh and Dyer (1959) and then quantified by the charring method (Marsh and Weinstein, 1966). Total lipids were expressed per biomass dry weight, per culture volume and per cell.

### Preparation and determination of fatty acids

A sample of 30–35 mg of dry biomass was extracted with 3.75 mL of chloroform : methanol (1 : 2). After a 20 min homogenization by vortexing, 1.25 mL of distilled water and 1.25 mL of chloroform were added. The chloroformic phase (containing lipids) was evaporated and dried under nitrogen. The extract was derivatized with 1.3 mL of 14 % boron trifluoride in methanol, incubated at 90 °C for 3 min and then 2 mL of hexane and 1 mL of distilled water were added. Samples were shaken and centrifuged at 3000 rpm for 10 min. The organic phase was removed and evaporated under nitrogen. The extract was dissolved in 100 µL of hexane, and 2 µL of the dissolved extract were injected into a gas chromatograph (Claurus 600, Perkin Elmer) equipped with a SPB-PUFA column and an FID detector operated at 260 °C. A programme of 160–210 °C for 90 min was used. The peaks obtained were identified by comparing their retention times with those of a mix of standard fatty acids (‘PUFA-1, Marine Source’, Supelco, Cat. 47033) and individual standards of arachidonic acid, docosahexaenoic acid, eicosapentaenoic acid, linolenic acid and gamma-linolenic acid (Sigma Aldrich). Relative quantification was performed by estimating the area of each peak with respect to the sum of the total areas of all peaks.

## Results

### Strain identification and phylogeny

**Figure 1.**
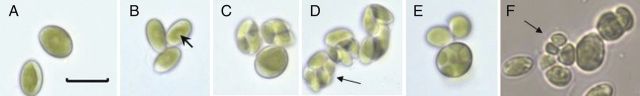
Light micrographs of the Coliumo strain, showing the morphology of vegetative cells (A, C and F) and sporangium (D and F, arrow). Sporangium with equal (C and D) and unequal sized autospores (F). Arrowhead = pyrenoid. Scale bar = 10 µm.

Under light microscopy, the Coliumo strain exhibits ellipsoidal or ovoid–ellipsoidal cells with more or less broadly rounded ends, surrounded by a thin cell wall. The chloroplast occupies most of the cell periphery except for the cell ends. The pyrenoid is spherical and more or less visible. Reproduction usually occurs by 2–4–8 autospores. Some sporangia exhibit autospores of different sizes (Fig. 1F). Cell sizes range from 4.5 to 10.5 µm long × 2.5 to 6.5 µm wide; sporangia: 5.7 to 10 µm in size (Fig. 1C–F). Cells of the Baker strain, on the other hand, are mainly spherical, 3.4–6.0 µm in diameter, with one parietal chloroplast and a distinct pyrenoid. Autospores are usually 2–6 within sporangia (5.6–7.5 µm in diameter) (Fig. 3).

**Figure 2.**
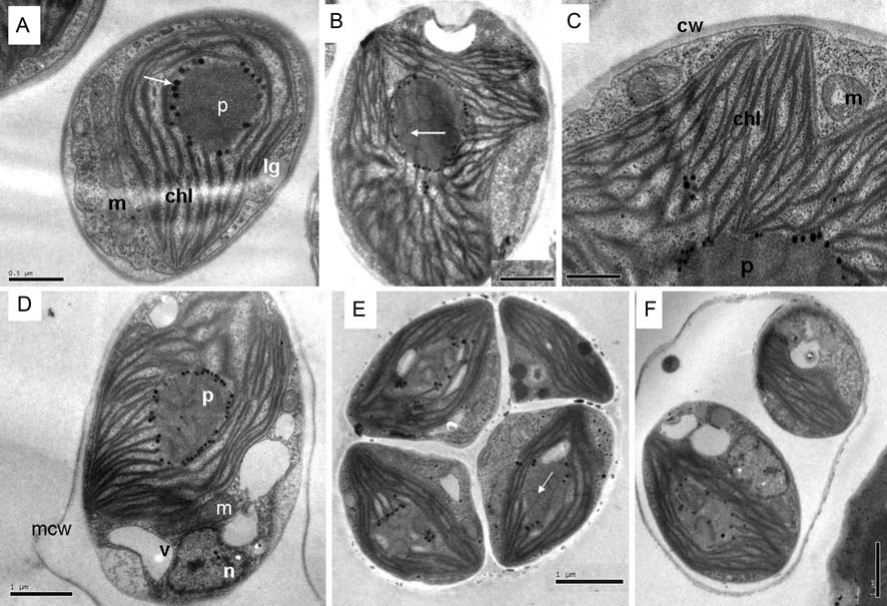
Transmission electron micrographs of the Coliumo strain. (A–D) Single cells showing the chloroplast (chl) shape and the pyrenoid (p) ultrastructure. Many undulating single thylakoids penetrate into the pyrenoid matrix (B and E, arrow). Note the presence of pyrenoglobuli around the pyrenoid (A, arrow and D). (E and F) Sporangia with four and two autospores. cw, cell wall; lg, lipid globule; m, mitochondrion; mcw, mother cell wall; n, nucleus; v, vacuole. Scale bars = 1 µm in all images, except in A and C = 0.5 µm.

Under TEM, cells of the Coliumo strain display either an ovoid or ellipsoidal shape (3.8–8.2 µm) (Fig. 2E and D). They contain a large saucer-shaped or irregularly undulated lobe at the margin of the chloroplast occupying most of the cell surface (Fig. 2A, B and D). A rounded or sometimes ovoid pyrenoid is present in the chloroplast. The pyrenoid, 1.1–2.3 µm in size, is surrounded by thylakoids. A few irregular or undulating thylakoids singly penetrate the pyrenoid matrix which never appears to be limited by a starch sheath (Fig. 2B, D and E). Starch is present, sometimes, in the form of small granules among the thylakoids. In addition, the pyrenoid of this strain is usually surrounded by a series of more or less electrodense osmiophilic globules (=pyrenoglobuli) (Fig. 2A, C and D). Occasionally, they have been observed inside the matrix associated with thylakoid outlines. The cell wall is electron transparent or slightly electro-dense, 38–52 nm thick.

**Figure 3.**
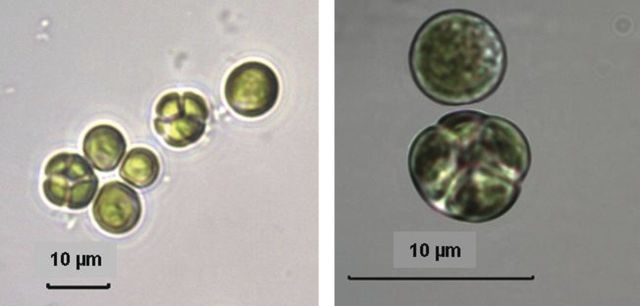
Light micrographs of the Baker strain, showing vegetative and sporangial cells.

**Figure 4.**
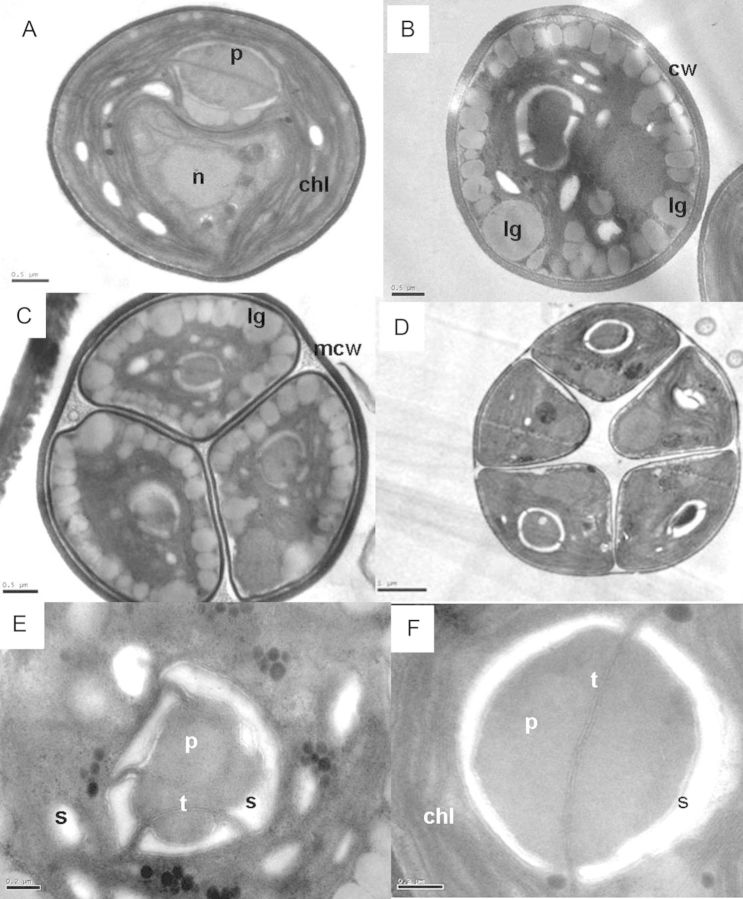
Transmission electron micrographs of the Baker strain. (A) Section of a vegetative cell showing the distribution of major organelles. (B) Section of a cell with many lipid globules (lg) lined up in the cell periphery. (C and D) Sporangia with autospores. Note in (C) the presence of lipid globules in each autospore. (E and F) Details of the pyrenoid. The pyrenoid (p) is surrounded by starch sheaths (s) and usually a double thylakoid (t) transverse it as seen in (F). chl, chloroplast; n, nucleus; cw, cell wall. Scale bars in A, B and C = 0.5 µm; D = 1 µm, and E and F = 0.2 µm.

On the other hand, the Baker strain exhibits rounded cells with a cup-shaped chloroplast occupying more than half of the cell surface (Fig. 4A); usually one pyrenoid per cell, 1.6–1.8 µm in size, covered by a starch sheath usually of two concavo-convex plates and divided by a double thylakoid (Fig. 4F); small starch granules can also be seen among the thylakoids (Fig. 4A, B and E). The cell wall appears as an electro-dense layer 90–125 nm thick. Some cells are characterized by a great number of lipid bodies restricting the space occupied by the chloroplast. These lipid bodies were also observed in newly divided daughter cells (Fig. 4C).

**Figure 5.**
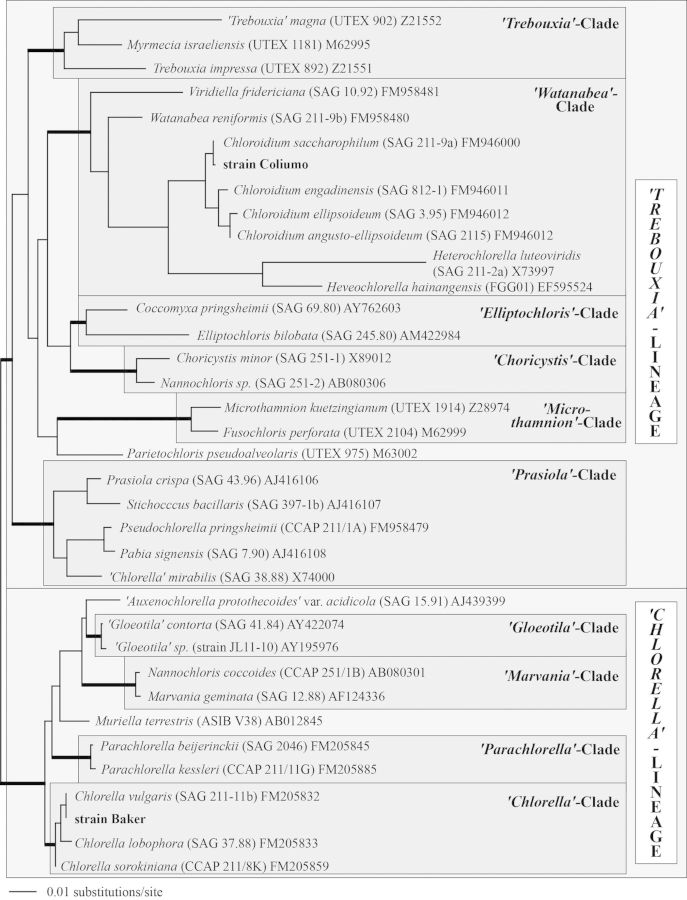
Molecular phylogeny of the Trebouxiophyceae based on SSU rDNA sequence comparisons. The phylogenetic tree (unrooted: midpoint rooting) shown was inferred by the maximum likelihood method based on a data set of 1747 aligned positions of 36 taxa using PAUP 4.0b10. For the analysis, the TIM model (base frequencies: A = 0.2467; C = 0.2259; G = 0.2794; T = 0.2480; rate matrix: A–C = 1.0000; A–G = 2.5989; A–T = 1.2872; C–G = 1.2872; C–T = 6.7566; G–T = 1.0000) with the proportion of invariable sites (*I* = 0.6138) and gamma distribution shape parameter (*G* = 0.5850) was chosen, which was calculated as the best model by Modeltest. Bootstrap values (>70 %) of the neighbour-joining (using the TIM+I+G model, 1000 replicates), maximum parsimony (1000 replicates) and maximum likelihood (using the TIM+I+G model, 100 replicates) methods were marked with branches in bold in the tree. Strain and accession numbers are given after the species name. The clade designation follows Pröschold *et al*. (2011).

The phylogenetic analyses of SSU rDNA sequences presented in Fig. 5 revealed that the Coliumo and Baker strains belong to *Watanabea* and *Chlorella* clades, respectively. The Coliumo strain is closely related to the authentic strain of *Chloroidium saccharophilum* (SAG 211-9a), while the Baker strain appears closely related to the authentic strain of *Chlorella vulgaris* (SAG 211-11b). The SSU rDNA sequence of the Coliumo strain differs only in one base compared with the strain SAG 211-9a, but in contrast contains two introns at positions 156 and 516 (see Darienko *et al*. 2010). The strains Baker and SAG 211-11b show two base differences in their SSU rDNA sequences.

**Figure 6.**
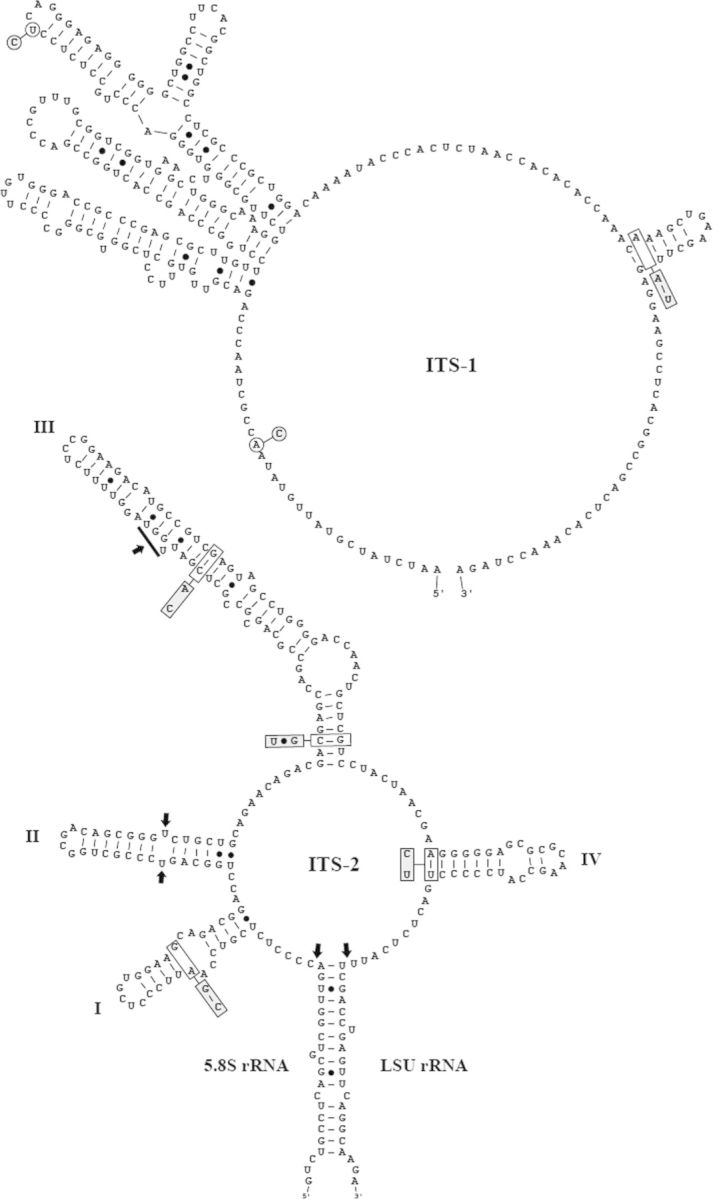
Comparison of the ITS-1 and ITS-2 rRNA secondary structures between the *C. saccharophilum* strains. The structure of the Coliumo strain is presented with the differences to SAG 211-9a, the authentic strain of this species, highlighted. Single base changes are marked in circles and base pair insertions are highlighted in grey boxes. The numbering of the helices followed the designation by Coleman and Mai (1997).

**Figure 7.**
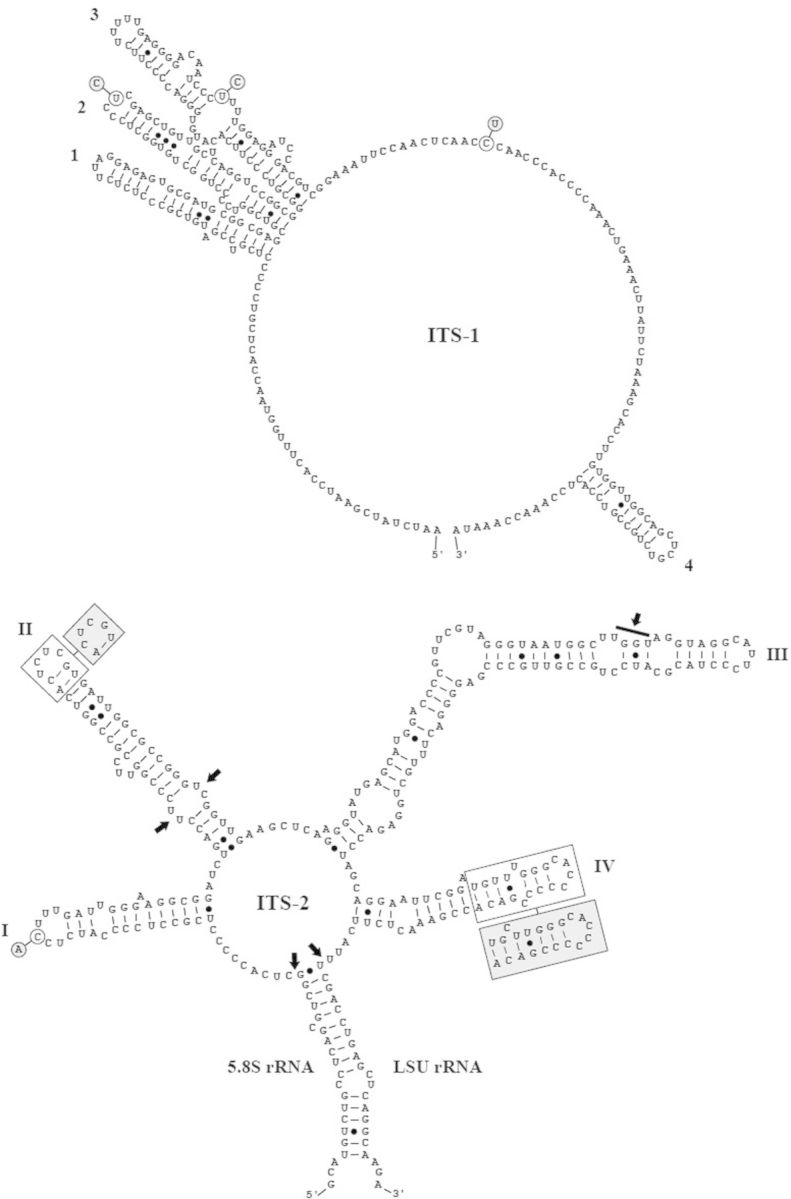
Comparison of the ITS-1 and ITS-2 rRNA secondary structures between the *C. vulgaris* strains. The structure of the Baker strain was presented with the differences to SAG 211-11b, the authentic strain of this species, highlighted. Single base changes are marked in circles and base pair insertions are highlighted in grey boxes. The numbering of the helices followed the designation by Coleman and Mai (1997).

To decide if both new isolates (Coliumo and Baker) belong to the type species of *Chloroidium* and *Chlorella*, respectively, we sequenced and compared the ribosomal internal transcribed spacer ITS-1 and ITS-2 sequences. The secondary structures of ITS-1 and ITS-2 of Coliumo and Baker are presented in Figs 6 and 7. The base changes compared with the authentic strains are highlighted in grey. The Coliumo strain shows three base differences in ITS-1 and four in ITS-2, including one hemi-compensatory base change (HCBC) in Helix III compared with SAG 211-9a. These base differences are only minor and clearly suggest that the Coliumo strain belongs to *Chloroidium saccharophilum*. A similar situation occurs for the Baker strain. The three base differences in ITS-1 and one base change and two different loops of Helices II and IV in ITS-2 compared with SAG 211-11b reveal that this strain belongs to *Chlorella vulgaris*.

### Lipid production

Table 1 shows the growth and lipid accumulation parameters of both strains grown under identical culture conditions. Except for the maximum cell density reached by the cultures (*N*_max_), all the parameters compared were significantly different between both strains. The Baker strain accumulated twice as much biomass as the Coliumo strain and it was much more oleaginous.

**Table 1:** Parameters of growth [maximum cell density (*N*_max_) and dry biomass per culture volume] and total lipid accumulation (total lipids, per dry biomass, per culture volume and per cell) of two Chilean *Chlorella*-like strains. Average values ± standard deviation of four replicates per strain are shown.

	*N*_max_(×10^6^ cells mL^−1^)	Dry biomass per culture volume	Total lipids per dry biomass	Total lipids per culture volume	Total lipids per cell
Strain		(mg mL^−1^)	(%)	(μg mL^−1^)	(pg cell^−1^)
Coliumo	6.29 ± 2.31	1.22 ± 0.21	14.37 ± 3.81	176.97 ± 59.29	28.33 ± 1.77
Baker	6.33 ± 1.10	2.11 ± 0.24	26.26 ± 3.41	559.7 ± 13.36	92.1 ± 3.38

With respect to the quality of the fatty acids, both strains accumulated similar proportions of saturated fatty acids, which are preferred for biodiesel production. On the other hand, almost 30 % of the total fatty acids in the Coliumo strain corresponded to the omega 3 alpha linolenic acid (ALA, C18 : 3 n-3) (Table 2), which has nutritional value.

**Table 2:** Fatty acid composition of two Chilean *Chlorella*-like strains. Average values ± standard deviation of four replicates per strain are shown. n.d., non-detected.

	Coliumo strain	Baker strain
Saturated fatty acids		
C12 : 0	n.d.	0.1 ± 0.1
C14 : 0	1.2 ± 0.3	1.3 ± 0.3
C16 : 0	22.5 ± 2.0	25.6 ± 3.0
C18 : 0	0.7 ± 0.4	1.2 ± 0.3
Sum (%)	24.4	28.1
Monounsaturated fatty acids		
C14 : 1 n-5	n.d.	0.4 ± 0.1
C16 : 1 n-7	0.5 ± 0.1	2.5 ± 0.4
C18 : 1 n-9	16.8 ± 2.3	21.3 ± 6.1
C20 : 1 n-11	0.3 ± 0.1	n.d.
Sum (%)	17.6	24.2
Polyunsaturated fatty acids		
C18 : 2 n-6	28.0 ± 1.8	12.7 ± 0.6
C18 : 3 n-6	n.d.	35.0 ± 5.3
C18 : 3 n-3	29.7 ± 3.8	n.d.
Sum (%)	57.7	47.7

## Discussion

According to the work of Ikeda and Takeda (1995), and based on the pyrenoid ultrastructure, the Baker strain corresponds to the *Chlorella* species which belong to the glucosamine-type group: *C. vulgaris*, *C. lobophora*, *C. sorokiniana* and *C. kessleri* (now *Parachlorella kessleri*; Krienitz *et al*. 2004). The thylakoid that penetrates into the pyrenoid matrix is uniformly double layered in all four taxa, as in the Baker strain. According to Takeda (1991, 1993), there are differences among these four species in cell wall characteristics [ruthenium red stainability (plus or minus) and anisotropy (plus or minus)]. Considering that the Baker strain under study was not subjected to cell wall characterization, and since *C. vulgaris* and *C. lobophora* shared these two attributes (rutheniun red: plus; anisotropy: minus), its identification as *C. vulgaris* was sustained on molecular data. Even though the 18S rDNA gene in the Baker strain has two introns which are missing in the authentic *C. vulgaris* species, the closely similar sequences in the ITS-1 and ITS-2 region confirmed its *C. vulgaris* identity. On the other hand, the Coliumo strain, and based also on pyrenoid ultrastructure (Ikeda and Takeda 1995), corresponds to the *Chlorella* species which belong to the glucan-type group, and specifically to *C. saccharophila*. The pyrenoid in the Coliumo strain is almost identical to the pyrenoid of *C. saccharophila* SAG 211-9a (fig. 4 of Ikeda and Takeda 1995). These features include the lack of a starch sheath around the pyrenoid that is surrounded by thylakoids. Many of the undulating single thylakoids penetrate the matrix of the pyrenoid which is usually surrounded by a series of more or less electrodense osmiophilic globules (=pyrenoglobuli) (Fig. 2A, C and D). Darienko *et al*. (2010) transferred all the *Chlorella*-like strains related to *C. saccharophila* to the genus *Chloroidium* Nadson and amended its identification by utilizing strain SAG 211-9a as the authentic strain of the type species, *Chloroidium saccharophilum* (Krüger) Darienko *et al*. (2010). Another attribute in common with *C. saccharophila* 211-9a (now *Chloroidium saccharophilum*) is the unequal size of the autospores during sporogenesis (see Fig. 1F). The molecular data confirmed the identity of the Coliumo strain as *Chloroidium saccharophilum* based on 18S rDNA and ITS-1 and ITS-2 sequence similarities.

Differences detected in growth parameters and the quantity and quality of lipids of both strains (Tables 1 and 2) can be explained by the taxonomic distance between both strains, which belong to different genera, as was determined in this research.

The value of the parameters ‘total lipid content per dry biomass’ and ‘total lipids per culture volume’ depends on the applications to which the microalgal biomass will be destined. A biomass with a high total lipid content per dry biomass is preferred if lipids need to be extracted because fewer purification steps are needed from a lipid-rich biomass. On the other hand, if crude biomass can be used directly as the lipid source, total lipids per culture volume is a more important parameter to consider at the commercial level due to the high costs associated with the harvesting process from diluted cultures. In this work, the Baker strain was the most oleaginous when expressed as both parameters (Table 1).

Griffiths and Harrison (2009) summarized the published information on lipid productivity for biodiesel production of many microalgal species, including *C. vulgaris*. From the literature, this species exhibited an average of 25 % lipids per dry weight and a lipid productivity of 26 mg L^−1^ day^−1^ under nutrient-replete conditions (considered non-inductive for neutral lipid accumulation, the type preferred for biodiesel production). The strain of *C. vulgaris* included in this research (Baker strain) had 26 % lipids per dry weight and a lipid productivity of 33 mg L^−1^ day^−1^, which fits well with the published values.

On the other hand, the strain of *Chloroidium saccharophilum* (Coliumo strain) was less oleaginous, but accumulated the nutritionally valuable omega-3 polyunsaturated fatty acid ALA (18 : 3 n-3). Alpha linolenic acid is the only omega-3 fatty acid considered to be essential for animals; its health benefits include cardioprotective effects, modulation of the inflammatory response and positive effects on central nervous system function, although these properties have been frequently overlooked (Stark *et al*. 2008). Unfortunately, there is no published information about the biochemical composition of *C. saccharophilum* to contrast our results; however, the quantity and quality of lipids accumulated by this species make it a good candidate for animal nutrition.

According to the results obtained in this work, *Chlorella vulgaris* (Baker strain) would be appropriate as a raw material for biodiesel production, while *Chloroidium saccharophilum* (Coliumo strain) would be more appropriate for animal nutrition. Nevertheless, it is important to consider that, in this research, no optimization of culture conditions was performed for any of the strains, which could significantly (and differentially) increase the productivity of total lipids and improve their quality for fuel production and/or animal nutrition.

## Conclusions

The use of ultrastructural characters (i.e. pyrenoid, location of starch grains and thylakoids) together with molecular data (ITS-2 secondary structure and phylogeny based on 18S rDNA sequences) allowed the identification, at genus and species level, of the two *Chlorella*-like strains under study: *Chloroidium sacharophyllum* (Coliumo strain) and *Chlorella vulgaris* (Baker strain).

*Chlorella vulgaris* (Baker strain) appeared to be suitable as a raw material for biodiesel production, while *Chloroidium saccharophilum* (Coliumo strain) would be more appropriate for animal nutrition. However, in this research, no optimization of culture conditions was performed for any of the strains, which could significantly (and differentially) increase the productivity of total lipids and improve their quality for fuel production and/or animal nutrition. Research is now in progress to test both strains under lipid-inductive laboratory conditions.

## Accession Numbers

The sequences were deposited in GenBank.

Submission # 1600175: 18S rRNA gene (partial), ITS-1, 5.8S rRNA gene and ITS-2 sequences of the *Chlorella vulgaris* strain (Baker strain).

Submission # 1600176: 18S rRNA gene (partial), ITS-1, 5.8S rRNA gene and ITS-2 sequences of the *Chloroidium saccharophilum* (Coliumo strain).

## Sources of Funding

This work was funded by ‘Fondo de Fomento al Desarrollo Científico y Tecnológico’ (FONDEF) Projects # D07I-1063 and # D07I-1017.

## Contributions by the Authors

M.A.G. was in charge in of obtaining the electron microscopy data in collaboration with Y.P. and P.A., and drafted the manuscript. T.P. did the sequence and phylogenetic analysis of *C. saccharophilum* and wrote that part of the manuscript. I.I. did the isolation and maintenance of the strains under culture conditions. P.I.G. and Y.P. did the sequence of *C. vulgaris* and were in charge of getting the growth and the lipid data, writing that part and reviewing the whole manuscript.

## Conflicts of Interest Statement

None declared.
